# The plantarward oblique Chevron osteotomy: an optional method to treat hallux valgus with painful plantar callosities

**DOI:** 10.1038/s41598-019-53479-6

**Published:** 2019-11-22

**Authors:** Jialiang Guo, Shiji Qin, Fengqi Zhang, Weichong Dong, Zhiyong Hou, Yingze Zhang

**Affiliations:** 1 Department of Orthopaedics, The Third Hospital of Hebei Medical University, Shijiazhuang, P.R. China; 2 Chinese Academy of Engineering, Beijing, P.R. China; 3 Department of pharmacy, The Second Hospital of Hebei Medical University, Shijiazhuang, P.R. China

**Keywords:** Trauma, Reconstruction

## Abstract

Hallux valgus (HV) is a foot deformity that can be treated with Chevron osteotomy, and a modified plantarward oblique osteotomy has been proposed in recent years. However, no research has focused on the correctional power of the osteotomy. The aim of this study was to examine the character of this plantarward oblique Chevron osteotomy (POCO) and to determine the rationale of this method.Radiographs and clinical data from 65 HV patients (77 feet) with painful callosities were evaluated. The intermetatarsal angle, hallux valgus angle, and relative height of the second metatarsal were measured, and a valid width of the first metatarsal was proposed. A visual analogue scale (VAS) and the American Orthopaedic Foot and Ankle Society hallux-metatarsophalangeal interphalangeal scale (AOFAS) were used to evaluate the patients’ clinical results.There were significant differences in the HVA and IMA. The decline in the height of the second metatarsal was positively related to the decline in the height of the first metatarsal, but the changes were smaller for the first metatarsal. Painful callosities disappeared in 77 feet, 4 (5.1%) patients had no pain but a remaining plantar callosity, and 2 (2.6%) patient had relieved pain with a plantar callosity after follow-up. The VAS scores improved from 8.58 ± 0.50 to 1.96 ± 0.75 points after the operation (p < 0.001). Significant differences were demonstrated in the AOFAS scores (65.81 ± 4.05 vs 87.88 ± 3.41, p < 0.001). The modified POCO prevents the dorsal migration of the metatarsal head, preserves other lesser metatarsals and provides an opportunity for patients who may possibly need additional future deformity correction. Therefore, POCO is a safe and effective method to treat hallux valgus and offers the superior potential benefits of correction and transfer metatarsalgia.

## Introduction

Hallux valgus (HV) is a foot deformity that affects 30% of people and causes pain and discomfort^1^. A V-shaped Chevron osteotomy is the treatment method used in mild or moderate hallux valgus patients without first metatarsophalangeal degeneration, but patients are sometimes unsatisfied with the results, mainly due to subsequent displacements, avascular necrosis or recurrences requiring a longer period of rehabilitation and transfer metatarsalgia caused by predisposing dorsal angulation^2^. To improve the effect of this treatment method, different variants have been proposed over time. Furthermore, painful plantar callosities under other lesser metatarsals are commonly associated with HV due to the reduced function of the plantar aponeurosis and the shortened length of the metatarsal^3^, and they often persist and develop under the second or other metatarsal head after surgery for hallux valgus, at a rate reported to be from 48% to 84%^4^.

Weil osteotomy is the most widely used osteotomy option to improve metatarsalgia^5^. However, the consensus regarding operative treatment for metatarsalgia with this osteotomy remains controversial. Mann found that 63% of painful callosities were resolved without the need for additional metatarsal osteotomy^6^. Lee also considered that painful plantar callosities under the lesser metatarsals in patients with HV could be treated by hallux valgus correction alone^4^. The mechanism was thought to be the restored alignment of the first ray and improved plantarflexion force.

Chung reported a modified scarf osteotomy (15 to 20 degrees of plantarward oblique osteotomy) that induced plantar angulation of the metatarsal head after lateral translation and stated that it reduced second transfer metatarsalgia^7^. In the traditional method, the direction of the Chevron osteotomy was recommended to be vertical to the plane after eliminating the osteophyte. Choi used the oblique method with Chevron osteotomy and found that there was a comparable effect with the modified scarf in correcting the height change in the second metatarsal head^8^. However, no research has focused on the relationship between the theoretical decline distance of the first metatarsal and the actual height changes of the second metatarsal head or the correctional power of the osteotomy. The aim of this research was to study the character of this plantarward oblique Chevron osteotomy (POCO) and to determine the rationale of this method.

## Materials and Methods

In this study, radiographs and the clinical data of HV patients with painful callosities were evaluated between June 2016 and June 2018. All were treated by two experienced surgeons in our hospital. The follow-up time was greater than 1 year (16.3 ± 1.1 months). Patients with calcaneal osteotomy, rheumatoid arthritis, gout or a history of trauma were excluded. All the methods were performed in accordance with the regulations and approved by the ethics committee of the Third Hospital of Hebei Medical University. Informed written consent was obtained from all subjects.

Hallux valgus with symptoms such as difficulty in wearing shoes, hallux valgus angle (≥20 degrees), IMA (>11 degrees), and an incongruent first metatarsophalangeal joint was indicated for hallux valgus surgery.

Metatarsalgia was recorded if the subject felt pain when walking with bare feet. The plantar callosity was evaluated with the method proposed by Nakagawa (grade 0: no plantar callosities were observed; grade 1: callosity without pain; grade 2: painful callosity under 1 joint; and grade 3: painful callosity under 2 or more metatarsal heads). All patients in the study had painful plantar callosities. Under the second metatarsal heads, 89 plantar callosities were observed. Among them, 7 callosities and 5 callosity were located under the third and fourth metatarsal heads, respectively, along with the second metatarsal heads. All patients complained of pain when walking, and no causes for the plantar callosities under the lesser metatarsal heads were found.

### Operative technique

**Figure 1:**
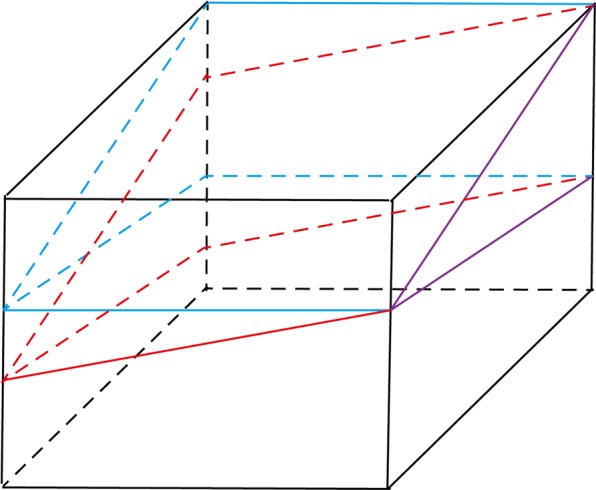
The comparison of the traditional Chevron and modified Chevron osteotomies. (**A**) The purple lines represent the osteotomy direction of these two techniques. (**B**) The blue lines represent the traditional osteotomy perpendicular to the longitudinal axis of the first metatarsal. (**C**) The red lines represent the modified Chevron osteotomy along with some degrees (θ) of plantarward obliquity.

**Figure 2:**
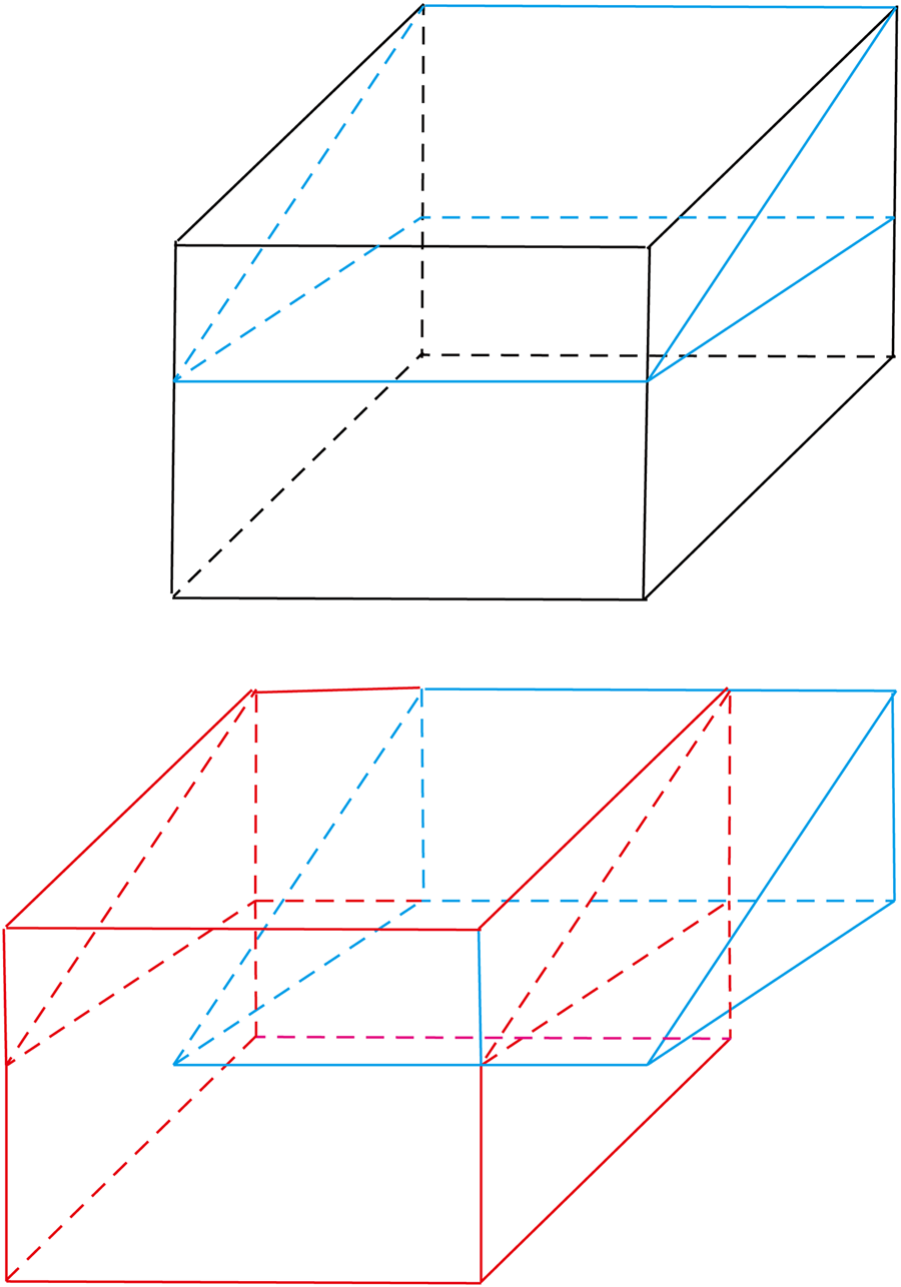
The mathematical model of the traditional Chevron osteotomy. The red part represents the distal section, and the blue part represents the proximal section after osteotomy.

**Figure 3:**
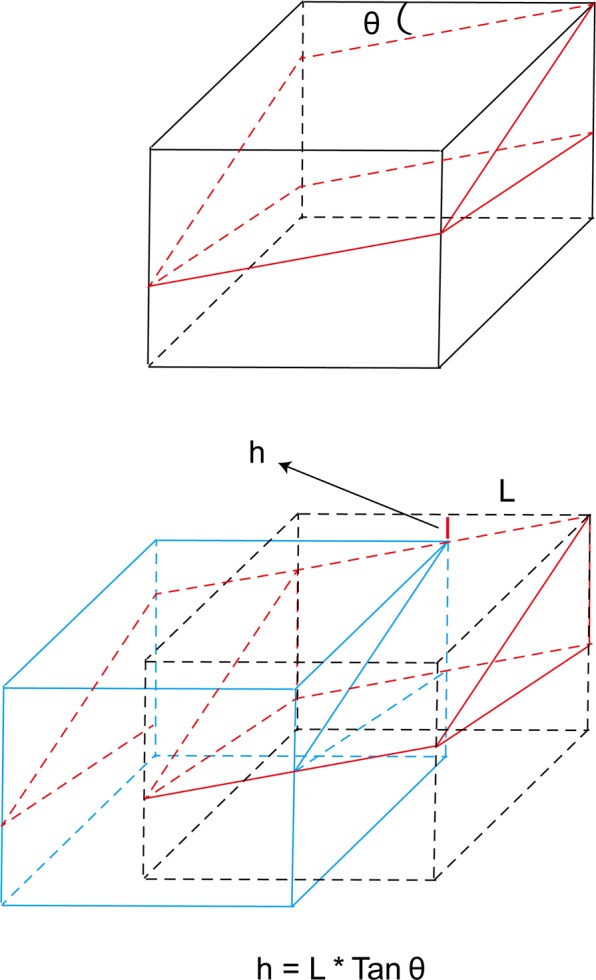
The mathematical model of the modified Chevron osteotomy used in our study. The red part represents the distal section, and the blue part represents the proximal section after the osteotomy. The θ was 20 degrees in our research, and the decreased height of the first metatarsal was calculated as h = L * Tan θ (θ = 20) = 0.36 L.

All procedures were performed by the same foot and ankle surgeon. Local anaesthesia was used for all the surgical cases. After the adductor hallucis tendon was detached from the proximal phalanx and the fibular sesamoid with a dorsal incision between the first and second metatarsal heads, the transverse intermetatarsal ligament and lateral capsule were also cut, and the ligament was released. After the joint capsule was dissected in an L-shape with a medial longitudinal incision, the medial osseous eminence was resected through a medial longitudinal incision with a saw. The modified Chevron osteotomy was performed as follows: the apex of the chevron osteotomy was located at the centre of the first metatarsal, and an osteotomy with an angle of 60 degrees along with 20 degrees of plantarward obliquity was performed (Figs. 1 and 2). The plantar cut was performed more horizontally than a traditional technique, resulting in a relatively longer plantar limb. Then, a guide wire was inserted at the apex and used to control the direction of the osteotomy, and each osteotomy was performed following the guidewire. To correct the deformity, the distal fragment was laterally translated (2–3 mm), and the first metatarsal head was also lowered (Fig. 3). One headless cannulated compression screw with a diameter of 2.7 mm was used to stabilize the osteotomy line. An Akin procedure was performed to correct any residual deformity in 19 patients. The skin was closed after the joint capsule was repaired.

After closure of the wound, 5-cm-wide elastic bandages were utilized to maintain the position of the great toe in a reduced position. After the surgical wound was stabilized, the patients were encouraged to walk with partial weight-bearing in orthopaedic post-op shoes after 14 days. The stitches were removed after two weeks, and the Kirschner wires were removed when the postoperative radiographs showed adequate bony union after surgery at approximately 6–8 weeks. Weight bearing on the whole foot was allowed 8 weeks postoperatively after the Kirschner wires were removed.

### Outcome measures

#### Radiographic measurement

**Figure 4:**
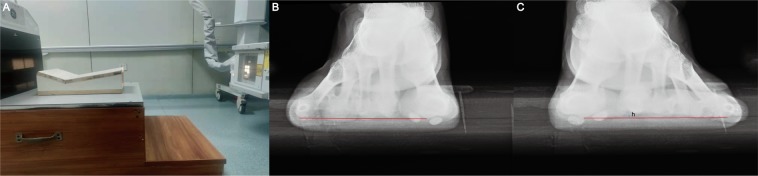
The height of the second metatarsal head. (**A**) A purposefully designed supporting plane was used to measure the height of the second metatarsal head. (**B**,**C**) The relative height of the second metatarsal head was defined as the perpendicular distance from the baseline, which was drawn between the lowest point of the fifth metatarsal head and first metatarsal head.

**Figure 5:**
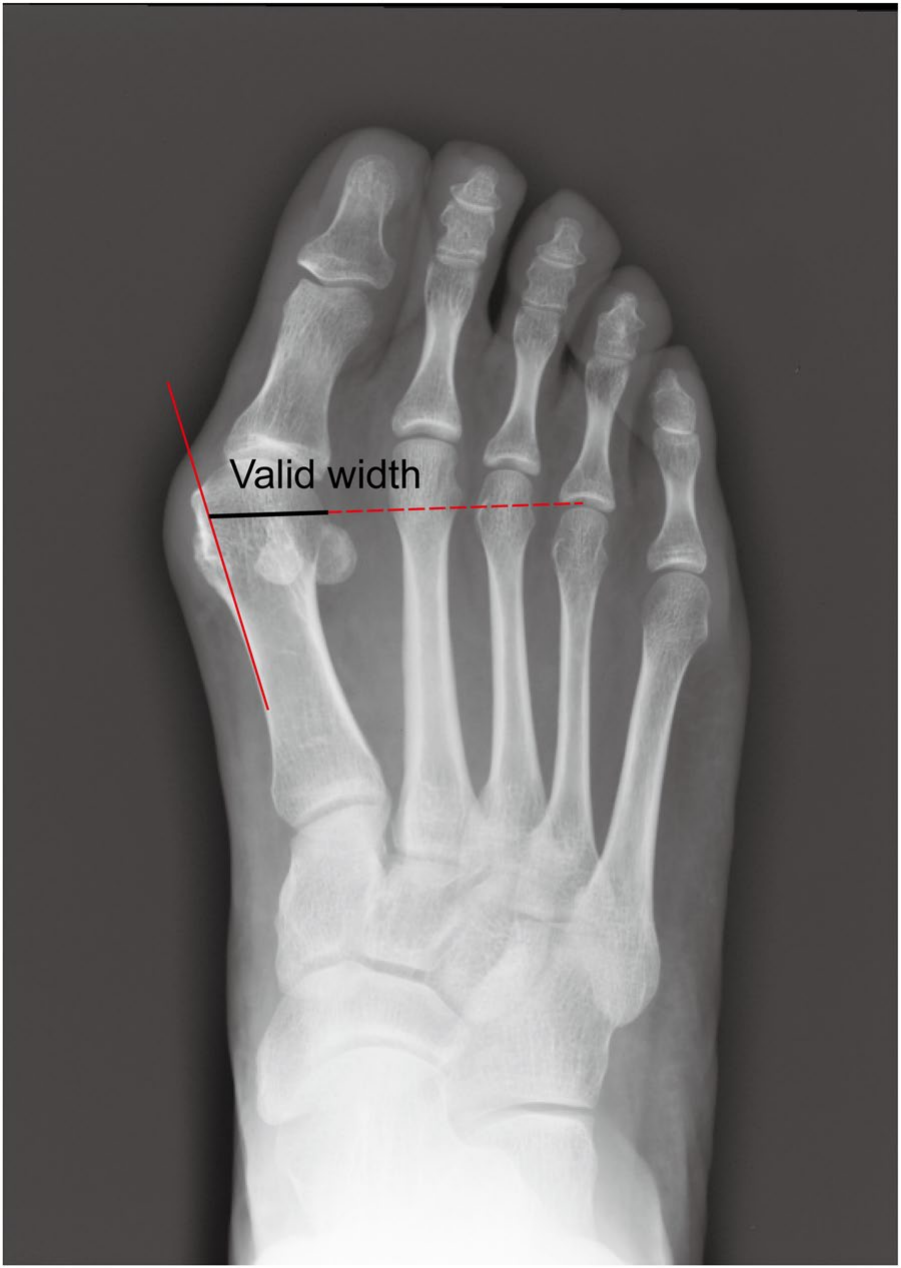
The measurement of the valid width of the first metatarsal. The osteotomy line of the medial eminence overlapped with the medial side of the metatarsal shaft. The line that was directed to the fourth metatarsophalangeal joint from the centre of the metatarsal head (1 cm proximal to the articular surface of the first metatarsal head) was drawn. The distance between the centre of the metatarsal head and the lateral cortices in the picture was the valid width of the first metatarsal in our study.

65 female and male patients (aged 27 to 73 years), or 77 feet, were evaluated. Standard weight-bearing anteroposterior radiographs were taken to evaluate the relative outcome measures for this study, such as the intermetatarsal angle (IMA) and hallux valgus angle (HVA). The relative height of the second metatarsal was also measured, and it was defined as the perpendicular distance of the second metatarsal head from the line that was connected to the lowest point of the first and fifth metatarsal heads (Fig. 4). The valid width of the first metatarsal was measured using the method shown in Fig. 5. The actual lateral transfer distance was recorded intraoperatively.

#### Clinical evaluations

A visual analogue scale (VAS) and the American Orthopaedic Foot and Ankle Society (AOFAS) hallux-metatarsophalangeal interphalangeal scale were used to evaluate the patients’ clinical results. Complications such as infection, delayed wound healing, malunion, and recurrence of hallux valgus were recorded.

### Statistical analysis

The means and standard deviations were used to illustrate continuous variables for the dependent parameters with SPSS 21 (SPSS Inc, Chicago, IL). The parametric t test and Pearson chi-square test were used to assess the differences between pre- and postoperation. *P < *0.05 indicated that there was a statistically significant difference. The correlations of the continuous variables were analysed using the Spearman correlation model.

## Results

### Radiographic outcomes

**Figure 6:**
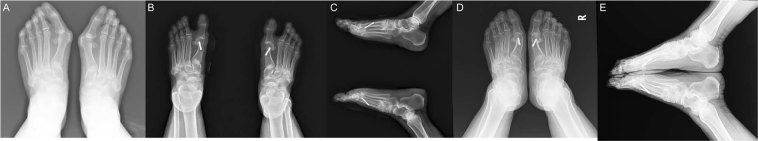
A patient with HV was followed-up. (**A**) The anterior-posterior view of the feet preoperatively. (**B**) The anterior-posterior view after the operation. (**C**) The lateral view after the operation. (**D**) The anterior-posterior view at the end of follow-up after 1 year. (**E**) The lateral view at the end of follow-up after 1 year.

The HVA improved from 29.42 ± 6.46 degrees to 14.32 ± 2.80 degrees postoperatively (*P* < 0.001), and there were also significant differences in the IMA (13.77 ± 1.32 vs 7.45 ± 1.00, *P* < 0.001) (Table 1, Fig. 6). The valid width of the first metatarsal was 16.32 ± 1.41 mm. The height of the second metatarsal was shown to have significant changes after the osteotomy, and the preoperative height was significantly lower than the postoperative height. The increase in the height of the second metatarsal was positively related to the decline in the height of the first metatarsal, although the increasing range was smaller than with the first metatarsal (Table 2, r = 0.626, *P* < 0.001). The lateral translation distances were all smaller than the 50% valid width of the first metatarsal.

**Table 1:** The measurement of relative parameters.

	Preoperative	Postoperative	*P*
HVA	29.42 ± 6.46	14.32 ± 2.80	<0.001
IMA	13.77 ± 1.32	7.45 ± 1.00	<0.001
Second MT head height, mm	0.05 ± 0.76	0.77 ± 0.73	<0.001
Valid width of the first MT, mm	16.32 ± 1.41	—	
Planed lateral transfer width, mm	2.69 ± 0.38	—	
Calculated decline height of the first MT, mm	0.97 ± 0.14	—

**Table 2:** The correlations of Second MT head height and calculated decline height of first MT head.

	Δ Second MT head height, mm	Calculated decline height
Δ Second MT head height, mm	0.97 ± 0.14	—
r	—	0.626
p	—	<0.001

### Clinical outcomes

There were no complaints of pain under the first metatarsal head after lowering in this study. All the patients in our research had a painful plantar callosity before surgery. Among the patients, painful callosities disappeared in 71 feet, 4 (5.1%) patients had no pain but a remaining plantar callosity, and 2 (2.6%) patients had relieved pain with a plantar callosity after follow-up. The VAS scores improved from 8.58 ± 0.50 to 1.96 ± 0.75 points after the operation (Table 3, *P* < 0.001). Significant differences were demonstrated in the AOFAS scores (65.81 ± 4.05 vs 87.88 ± 3.41, *P* < 0.001). Loss of correction was not observed in the study, and no infection, delayed wound healing, malunion, or recurrence of hallux valgus were found after follow-up. Two patients had first metatarsophalangeal joint stiffness, but no revision was needed.

**Table 3:** The results of clinical evaluations.

	Preoperative	Postoperative	*P*
VAS (0–10)	8.58 ± 0.50	1.96 ± 0.75	<0.001
AOFAS (0–100)	65.81 ± 4.05	87.88 ± 3.41	<0.001

## Discussion

The objective of surgical treatment of HV is to correct the deformity and improve patients’ symptoms. However, painful plantar callosities or metatarsalgia associated with hallux valgus may persist or even develop after surgery. To solve the problem, the modified POCO was used in this study; 71 of 77 feet with painful plantar callosities recovered, and other patients had a plantar callosity without pain or relieved pain. Therefore, it is concluded that POCO is a safe, effective method to treat hallux valgus and offers superior potential benefit of correction and transfer metatarsalgia. In our opinion, hallux valgus is a deformity that occurs in three different planes (coronal, sagittal and axial planes), and the modified POCO used in our research can solve the deformities in the coronal (traditional osteotomy, medial-lateral direction) and sagittal planes (plantar-modified osteotomy, dorsal-plantar direction). The study enriches our understanding of the treatment of HV with modified POCO and contributes to the treatment of hallux valgus with painful plantar callosities.

When hallux valgus is moderate or severe, the second toe may be elevated and bestridden over the first metatarsal. Then, excessive dorsiflexion may result in a increasing of pain of the second metatarsal head (proximal phalanx presses on the head in unnormal metatarsophalangeal joints) followed by possible transfer metatarsalgia. Not all painful plantar callosities that can be treated with surgery or conservative treatment are located under the second metatarsal head. However, a consensus regarding operative treatment for transfer metatarsalgia with a multifactorial aetiology has not been reached. Some surgeons support the lesser metatarsal osteotomy due to the possibility of improved, persistent, or developed metatarsalgia after surgery for hallux valgus. Yamamoto reported that only 48% recovered without a lesser metatarsal osteotomy in the treatment of HV^9^. In contrast, others think that it is not necessary to have another operation on the lesser metatarsal. Lee proposed that painful plantar callosities under the lesser metatarsals in patients with HV could be improved by hallux valgus correction alone without lesser metatarsal osteotomy^4^. Mann also reported a 63% complete recovery rate for painful plantar callosities after hallux valgus correction without lesser metatarsal osteotomy^6^. Consistent with Mann’s opinion, we consider that osteotomy of the lesser metatarsal head not only increases the financial burden if all the callosities are treated with osteotomy but also poses a considerable challenge for orthopaedic surgeons. Generally, painful plantar callosities result from the deformity of the sagittal plane. Most previous methods have included a single coronal plane osteotomy, which is performed on the plane that is perpendicular to the cut plane of the medial eminence, and the excision occurs parallel to the medial side of the metatarsal shaft. The modified POCO in our research is actually a biplanar osteotomy that decreases the height and increases the pressure under the first metatarsal head, and it reserves the opportunity for another operation (Weil osteotomy to the metatarsal head) for patients when the pain is not relieved after the indirect intervention used in the study fails.

The valid width of the first metatarsal was measured, and the lateral transfer distance was controlled at 2–3 mm intraoperatively, which had no effect on the stability after the osteotomy (the maximum distance of lateral transfer was 50% of the valid width). In addition, there was a positive correlation between the changes in the second metatarsal height postoperatively and the decline in the height of the first metatarsal, although the actual changes in the second metatarsal were smaller than the decline of the height in the first metatarsal. The decreased changes in the second metatarsal height may be explained by the restricting effect of soft tissue around the first metatarsal. Furthermore, it was demonstrated that the majority of painful plantar callosities were alleviated or disappeared without a lesser metatarsal osteotomy. It was noticed that the callosities could be improved along with the hallux valgus deformity even if the improvement in some patients with a negative second metatarsal head after the operation was attributed to the redistribution of plantar pressure or to the decrease in pressure under the lesser metatarsal heads. Choi reported that the incidence of a persistent painful plantar callosity under the second metatarsal head after surgery was high (25.9%) compared with Scarf and proximal osteotomies^8^. However, the results of our study were different in that painful plantar callosities were alleviated, and the reason could be that although the method used was similar, most of the patients enrolled had more severe painful preoperative metatarsalgia than Choi’s patients, so they were more inclined to have benefited from POCO. Without the controlled lateral transfer, the correctional power of POCO can be increased after evaluating the width of the first metatarsal carefully. However, there are also disadvantages if the decline in the width of the head is too large, and excessive lowering of the metatarsal head is considered to increase the joint pressure (between the first metatarsal and medial sesamoid bone) so that pain or arthritis may occur postoperatively, but arthritis was not observed in our study, which shows that the decline in the width of the metatarsal head was restricted due to a lateral transfer distance (2–3 mm). Furthermore, the volume of the distal part after POCO was decreased (Fig. 3), so the weight-bearing time was delayed compared with other studies.

In some studies, transfer metatarsalgia was improved by maintaining the length of the first metatarsal^10,11^. Therefore, osteotomy correcting the hallux valgus deformity with minimized shortening of the first metatarsal was recommended^12^. Nevertheless, even if the osteotomy direction is perpendicular to the first metatarsal longitudinal axis, the shortening does occur rather frequently (the bone loss from sawing)^8,12,13^. Choi considered transfer metatarsalgia to be treated by increasing the first metatarsal length or decreasing the second metatarsal length (only preferred when first metatarsal lengthening was limited by soft tissue)^8^. In addition to the method of changing length, Guler proposed that distal oblique metatarsal osteotomy provides plantar displacement of the distal fragment of the first metatarsal to compensate for shortening of the metatarsal^12^. Consistent with Gluer, the method used in our study also resulted in plantar displacement. Furthermore, the POCO could be optimized by maintaining or increasing the length of the first metatarsal. If the IMA or HVA is corrected without osteotomy (which is impossible), the length is preserved, and no complications will occur. However, if the deformity is corrected by osteotomy, the valid length of the first metatarsal compared to the normal limb decreases due to its changed axis, especially for patients with severe varus before osteotomy in the process of deformity. Therefore, a small or no change in the first metatarsal bone length after osteotomy is not ideal, and the increased length of the first metatarsal to some extent is more reasonable to rebalance the pressure under the metatarsal. The increased length of the first metatarsal can be realized with the modified POCO when the direction of the osteotomy is adjusted in the axial planes (the deformity in the distal-proximal directions such as the length of the metatarsal). To increase the length after the lateral transfer, the direction of the osteotomy should point to the distal part of the first metatarsal (distal to the centre of the fourth metatarsophalangeal joints). However, the simultaneous manipulation of the direction in plantar-dorsal and distal-proximal directions is difficult, and the measurement of the height of the metatarsal is also difficult due to the overlap in radiographs. In this study, the pain under the second metatarsal head was relieved, and the axial deformity could also be corrected partly with Akin, which was safe and easy to conduct, so increasing the length of the first metatarsal was not performed in our study. However, in a following study, more work will be performed to study the feasibility of a creative triplanar osteotomy method in severe HA patients.

The limitations of the study were that the number of patients enrolled was limited, and the study focused only on short period outcomes. POCO might increase the risk of osteonecrosis, but the short follow-up period demonstrated that it was safe and may benefit from the relative horizontal and long plantar osteotomy lines. Furthermore, the volume of the distal part after the osteotomy was reduced, and the effect on the stability of HA should be evaluated in future research. However, the technique used in our study was still feasible and effective in guiding the treatment of HA with painful plantar callosities.

In conclusion, not all patients had a lower position of the first metatarsal compared with the second one after the modified POCO, but modified POCO was still a reliable technique to relieve or avoid the occurrence of transfer metatarsalgia. The valid width of the first metatarsal was first proposed and reminded surgeons that there is more potential capacity for correcting HV deformities with POCO. Furthermore, the height of the second metatarsal can be used to guide the lateral transfer distance of the first metatarsal preoperatively, especially for those patients with crossed toes. The modified POCO prevents the dorsal migration of the metatarsal head and preserves other lesser metatarsals, providing an opportunity for possible future deformity correction in patients. Therefore, POCO is a safe and effective method to treat hallux valgus and offers superior potential benefits of correction and transfer metatarsalgia.

## References

[1] Hecht PJ, Lin TJ (2014). Hallux valgus. Med Clin North Am.

[2] de Las Heras-Romero J (2019). A new minimally extended distal Chevron osteotomy (MEDCO) with percutaneous soft tissue release (PSTR) for treatment of moderate hallux valgus. Foot (Edinb).

[3] Okuda R, Kinoshita M, Morikawa J, Jotoku T, Abe M (2001). Surgical treatment for hallux valgus with painful plantar callosities. Foot Ankle Int.

[4] Lee KB, Park JK, Park YH, Seo HY, Kim MS (2009). Prognosis of painful plantar callosity after hallux valgus correction without lesser metatarsal osteotomy. Foot & ankle international.

[5] Melamed EA, Schon LC, Myerson MS, Parks BG (2002). Two modifications of the Weil osteotomy: analysis on sawbone models. Foot Ankle Int.

[6] Mann RA, Rudicel S, Graves SC (1992). Repair of hallux valgus with a distal soft-tissue procedure and proximal metatarsal osteotomy. A long-term follow-up. J Bone Joint Surg Am.

[7] Chung JW, Jung HW, Chu IT (2008). Modified scarf osteotomy for hallux valgus with lesser metatarsalgia [in Korean]. J Korean Foot Ankle Soc.

[8] Choi JY, Suh YM, Yeom JW, Suh JS (2017). Comparison of Postoperative Height Changes of the Second Metatarsal Among 3 Osteotomy Methods for Hallux Valgus Deformity Correction. Foot Ankle Int.

[9] Yamamoto K, Imakiire A, Katori Y, Masaoka T, Koizumi R (2005). Clinical results of modified Mitchell’s osteotomy for hallux valgus augmented with oblique lesser metatarsal osteotomy. J Orthop Surg (Hong Kong).

[10] Toth K, Huszanyik I, Boda K, Rode L, Kellermann P (2008). The influence of the length of the first metatarsal on transfer metatarsalgia after Wu’s osteotomy. Foot Ankle Int.

[11] Barouk P (2014). Recurrent metatarsalgia. Foot Ankle Clin.

[12] Guler O, Yilmaz B, Mutlu S, Cerci MH, Heybeli N (2017). Distal Oblique Metatarsal Osteotomy for Hallux Valgus Deformity: A Clinical Analysis. J Foot Ankle Surg.

[13] Lee JY, Lee YS, Song KC, Choi KY (2015). Change in First Metatarsal Length After Proximal and Distal Chevron Osteotomies for Hallux Valgus Deformity. J Foot Ankle Surg.

